# Malignant behaviorial characteristics of CD133^+/−^ glioblastoma cells from a Northern Chinese population

**DOI:** 10.3892/etm.2012.747

**Published:** 2012-10-15

**Authors:** XIAOZHI LIU, LEI CHEN, ZHONGMIN JIANG, JUNFEI WANG, ZHIGUO SU, GANG LI, SHIZHU YU, ZHENLIN LIU

**Affiliations:** 1 Department of Neurosurgery, Tianjin Binhai Neurological Institute;; 2 Department of Pathology, The Fifth Central Hospital of Tianjin;; 3 Department of Neuropathology, Tianjin Neurological Institute, Tianjin Medical University General Hospital;; 4 Key Laboratory of Post-Trauma Neuro-Repair and Regeneration in the Central Nervous System, Ministry of Education;; 5 Tianjin Key Laboratory of Injuries, Variations and Regeneration of Nervous System, Tianjin, P.R. China

**Keywords:** glioblastoma, tumor stem cell, invasiveness, tumor recurrence

## Abstract

Following emergence of the tumor stem cell theory, the increasing number of related studies demonstrates the theory’s growing importance in cancer research and its potential for clinical applications. Few studies have addressed the *in vitro* or *in vivo* properties of glioma stem cells from a Han Chinese population. In the present study, surgically obtained glioblastoma tissue was classified into two subtypes, CD133^+^ and CD133^−^. The hierarchy, invasiveness, growth tolerance under low nutrient conditions and colony forming abilities of the tissue samples were analyzed. Additionally, the characteristics of tumor cells transplanted subcutaneously or re-transplanted into nude mice were observed. The results demonstrated that CD133^+^ glioblastoma cells derived from Han Chinese glioma specimens were more prone to primitive cell differentiation and more invasive than CD133^−^ glioblastoma cells, leading to increased tumor malignancy compared with CD133^−^ cells. The tumor formation rates of CD133^+^ and CD133^−^ cells in mice were 26/30 and 2/30, respectively. A comparison of tumor subtypes demonstrated that CD133^+^ glioblastoma cells had a lower incidence of cell apoptosis in the tumor tissue and higher protein expression levels of Oct4, Sox2, PCNA, EGFR, Ang2, MMP2 and MMP9 compared with CD133^−^ cells. Flow cytometry revealed that in the CD133^+^ and CD133^−^ glioblastoma cell-induced tumors, the percentage of CD133^+^ cells was 2.47±0.67 and 0.44±0.14%, respectively. The tumor formation rates following the re-transplantation of CD133^+^ or CD133^−^ tumors into nude mice were 10/10 and 4/10, respectively. These findings suggest that the CD133^+^ glioblastoma cell subpopulation has a stronger malignant cell phenotype than the CD133^−^ subpopulation and that its recurrence rate is increased compared with the primitive tumorigenic rate following *in vivo* transplantation.

## Introduction

Although surgery-based comprehensive therapy has greatly improved the treatment of gliomas, the prognosis of high-level gliomas remains poor (1–3). There is little evidence addressing the reasons for glioma recurrence following extensive tumor resection and post-surgical sequential treatments, such as radiotherapy and chemotherapy (4). The tumor stem cell theory has provided novel hypotheses for the further understanding of gliomas and related treatments.

According to the tumor stem cell theory, the majority of tumor cells are not tumorigenic; rather it is the tumor stem cells that determine tumor occurrence, development, metastasis and recurrence. Existing conventional treatments fail to diminish tumor stem cell numbers or functions and thus tumor recurrence is common (5–8). Tumor stem cells may be present in other locations far from the clinical lesions, and may form tumors through migration and seeding. Thus, they are difficult to detect and remove by conventional surgical resection. Tumor stem cells may also possess the capacity for immune escape (9) which may be significant in the establishment of tumor microcirculation (10). An understanding of the characteristics of tumor stem cells and the mechanisms associated with their ‘escape’ from clinical therapy is required, in conjunction with the use of new treatments based on the clearance and intervention of tumor stem cells, to advance the treatment of glioma.

In 2003, Singh *et al* were the first to identify a cell subpopulation with an unlimited proliferative potential and specific differentiation potential in medulloblastoma and glioblastoma multiforme. These cells possessed different molecular genetics and cell biology characteristics compared with common brain tumor cells since they expressed neural stem cell markers, such as nestin, Musashi-1, Bmi-1 and CD133. Additionally, these cells had an increased self-renewal and proliferation ability compared with neural stem cells (11). These cells were able to differentiate into tumor cells with the same phenotype as the original tumor *in vitro* and form tumors *in vivo* following transplantation. Tumor stem cells in the brain are resistant to radiotherapy and chemotherapy and consequently an increasing amount of glioma stem cell research has been carried out. However, there is controversy with regard to the sorting and identification criteria of brain tumor stem cells. For example, nestin was considered to be a specific brain tumor stem cell marker (12) but was later found to be expressed in progenitor cells during differentiation (13). CD133 is a transmembrane protein with a relative molecular weight of 120,000 Da and was initially used as a hematopoietic stem cell marker. CD133 is a common marker in neural stem cells, rather than a specific marker of brain tumor stem cells (14). However, CD133 may be useful for identifying brain tumor stem cell-specific markers and is considered to be important for the separation and purification of brain tumor stem cells, as well as in tumor development and prognosis.

In the present study, we aimed to conduct CD133^+/−^ cell selection in the glioblastoma tissues of 8 individuals from Northern China and to analyze the biological characteristics of the two cell subtypes through *in vivo* and *in vitro* observations. The results verified the high tumorigenicity and invasiveness of CD133^+^ tumor cells and demonstrated the limitations and deficiencies of using CD133 alone as a marker to distinguish tumor stem cells.

## Materials and methods

### Tissue specimens

Eight samples of glioblastoma tissue were obtained from patients admitted to the Department of Neurosurgery at the Fifth Central Hospital of Tianjin (Tianjin, China) between August 2009 and September 2011. All patients were Han Chinese individuals from the Beijing, Tianjin, Hebei Province and Shandong Province regions of Northern China. All patients and their family members agreed and signed a consent to enroll in the study.

### Tumor tissue digestion and detection of the CD133^+^ cell percentage

Glioblastoma blocks were rinsed twice with D-Hank’s solution on an ultraclean table and the tissue block was sheared into paste after vessels and necrotic tissue were eliminated. The paste was then digested with 0.25% trypsin (Invitrogen, Carlsbad, CA, USA) and cells were pipetted into a single-cell suspension. Digestion was terminated using 10% fetal bovine serum (Hangzhou Sijiqing Biological Materials Co., Ltd., Hangzhou, China). Tissues were filtered through a 30-*μ*m mesh and centrifuged at 1,000 rpm for 5 min. Following the removal of the supernatant, cells were collected and counted. Cells (1×10^6^) were suspended in 100 *μ*l phosphate-buffered saline (PBS), stained with isothiocyanate-labeled anti-CD133 antibody (Chemicon, Temecula, CA, USA) for 30 min in darkness and re-suspended with 1% paraformaldehyde (600 *μ*l). The percentage of CD133^+^ cells was analyzed using flow cytometry.

### Immunomagnetic separation of CD133 glioblastoma cells

Cells were suspended with PBE incubation solution (0.5% bovine serum albumin, 0.08% EDTA in PBS, pH 7.2) to a final concentration of 1×10^8^ cells in 0.5 ml, then incubated with anti-CD133 antibody (final antibody concentration 20 *μ*g/ml) at 4°C for 30 min and incubated with antibody-coated superfine magnetic beads (Miltenyi Biotec GmbH, Bergisch Gladbach, Germany) at 10°C for 15 min and suspended in 20 times the total volume of PBE solution. The separation column was installed into a magnetic field and pretreated with 0.5 ml PBE which was naturally eluted due to gravity. The incubated cell suspension was added to the separation column and naturally eluted Then 0.5 ml PBE was added to the separation column and naturally eluted. The column was rinsed twice and then separated from the magnetic field. The column was subsequently inserted into a new tube and 1–2 ml PBE was administered along the needle core to remove the CD133-positive cells. Simultaneously, negative cells were collected and the two types of cells were rinsed with medium.

### Immunocytofluorescence assay

CD133^+^ and CD133^−^ cells were prepared on cell slides and the coverslip was pre-coated with polylysine. Immunocytofluorescence was performed 12 h later to determine the expression levels of surface neural stem cell nestin (Santa Cruz Biotechnology, Inc., Santa Cruz, CA, USA), glial cell glial fibrillary acidic protein (GFAP, Chemicon) and neuron-specific enolase (NSE, Chemicon). Immunocytofluorescence procedures were performed as follows. Tissues were fixed with 4% paraformaldehyde at room temperature for 30 min, treated with 1% Triton X-100, washed with PBS for 3 min 3 times and blocked with goat serum for 30 min. After the serum was removed, cells were incubated with the appropriate dilution of antibody in a wet box at 4°C overnight. Cells were then incubated with FITC- or TRITC-labeled antibodies (1:100) in a wet box at 37°C for 45 min, followed by rinsing with PBS and deionized water. Sections were then mounted with Vectashield sealing agent and observed under a fluorescence microscope. Antibody dilutions were as follows: nestin (1:200), GFAP (1:500) and NSE (1:100).

### Measurement of cell invasiveness

Transwell (Chemicon, Temecula, CA, USA) upper chambers were pre-coated with a matrix adhesive (Sigma, St. Louis, MO, USA) and CD133^+^ and CD133^−^ glioblastoma cells were incubated in the upper chamber at a density of 1×10^4^ cells/well with each type of cell repeated in eight wells. Each well in the lower chamber was cultured with 600 *μ*l DMEM containing 10% fetal bovine serum. After 48 h, the upper chamber was removed and the non-adherent cells on the membrane surface were wiped away with a wet cotton swab. Following hematoxylin staining, tissue was mounted at 80°C and dried. The adherent cells on the membrane were observed under an inverted microscope.

### Measurement of tumor cell serum dependence

CD133^+^ or CD133^−^ glioblastoma cells were cultured in conditioned DMEM containing 10% fetal bovine serum and eight wells out of a 96-well culture dish were selected for trypan blue staining. The average number of living cells and the percentage of the total number of cells was calculated using light microscopy.

### Cell cloning

A 6-well plate was pre-coated with 0.7% agar (Zhongshan Biocorp.) and allowed to coagulate. Low-melting agarose (Zhongshan Biocorp.) working fluid at a final concentration of 0.35% was mixed with CD133^+^ and CD133^−^ glioblastoma cell suspensions and then added into a 6-well plate. Cells were coagulated in an incubator overnight and the following day 200 *μ*l full medium was added to each well. Three weeks later, 10 fields of vision were randomly selected under an inverted microscope and if there were >40 clones in these fields, the mean number of cells was calculated.

### Subcutaneous cell transplantation experiments in nude mice

A total of 60 C57BL/6 male thymectomized mice, weighing 80 g, were purchased from the Animal Breeding Center of the Academy of Military Medical Sciences of Chinese PLA, and maintained at the Animal Experiment Center of Tianjin Medical University, at 20–25°C and in 50±5% humidity (lot no. Beijing 0195). All experimental procedures were carried out according to the regulations and internal biosafety and bioethics guidelines of Tianjin Medical University and the Tianjin Municipal Science and Technology Commission. CD133^+^ and CD133^−^ glioblastoma cells were adjusted to a final concentration of 4×10^10^ cells/l. Mice were divided into two groups of 30 mice. The area between the right leg and abdominal cavity in the nude mice was disinfected with iodine and a single cell suspension was injected into the mice subcutaneously using a 50-*μ*l micro syringe. The needle was held in place for 1 min and then gradually withdrawn to prevent liquid return. After inoculation, mice were housed in a sterile barrier system at constant temperature (25±2°C) and humidity (45–50%). Tumor formation and growth were observed daily.

### Tumor tissue sampling and preparation

CD133^+^ and CD133^−^ glioblastoma cell-induced subcutaneous tumors were observed in 26 and 2 nude mice, respectively, and extracted on a super-clean table. Tumor tissue was divided into four sections of varying volumes according to the requirements of subsequent experiments, and used for frozen sections, protein extraction, digestion for preparation of a single cell suspension and tumor transplantation.

### In situ apoptosis analysis

Frozen sections were washed with PBS for 5 min twice, soaked in a semipermeable membrane for 5 min, and incubated with TUNEL (Beijing Zhongshan, China) labeling reaction mixture (25 *μ*l) in a wet box at 37°C for 60 min and then rinsed with PBS for 5 min 3 times. After the PBS was removed, each section was incubated with Hoechst 33258 (1:1,000; Santa Cruz) to counterstain cell nuclei. Cells were incubated in darkness for 10 min and rinsed with PBS and deionized water, then tissue was mounted with a fluorescence agent and observed under fluorescence microscopy. The cell apoptotic rate was calculated according to the following formula: (number of apoptotic cells/total cell number) × 100%.

### Western blot assay

A total of 40 *μ*g lysates were subjected to SDS-PAGE on 8% SDS-acrylamide gel. Separated proteins were transferred to PVDF membranes (Millipore, Bedford, MA, USA) and incubated with primary antibodies against Oct4 (1:1,000; Zhongshan Bio Corp.), Sox2 (1:1,000; Zhongshan Bio Corp.), PCNA (1:200; Zhongshan Bio Corp.), EGFR (1:1,000; Santa Cruz Biotechnology, Inc.), Ang2 (1:500; Chemicon), MMP2 (1:500; Zhongshan Bio Corp.) and MMP9 (1:200; Santa Cruz Biotechnology, Inc.) at 4°C overnight. The following day, cells were incubated with horseradish enzyme-labeled antibody (1:500) at 7°C for 2 h. A chemiluminescence reagent kit was used to develop the reaction and a Bio-Rad gel imaging system was used to determine the absorbance value which was analyzed with Quantity One software.

### Flow cytometry

After blood vessels and necrotic tissue in tumors were eliminated, tumor tissue was sheared into a paste, digested with 0.25% trypsin and pipetted repeatedly into a single-cell suspension. The digestion was terminated with 10% fetal bovine serum, cells were filtered with a 30-*μ*m mesh and centrifuged at 1,000 rpm for 5 min. Cells were counted following the removal of the supernatant, and 1×10^6^ cells were suspended in 100 *μ*l PBS, stained with isothiocyanate-labeled anti-CD133 antibody for 30 min in darkness and then resuspended in 600 *μ*l 1% paraformaldehyde. The percentage of CD133^+^ cells was detected by flow cytometric analysis.

### Tumor formation rate following in vivo transplantation

Tumor tissue was carefully dissected on a superclean table and fish-shaped tissues were harvested and cut with scissors into 1-mm^3^ sections which were stored in PBS for further use. The neck dorsal skin of nude mice was fixed with the left thumb and forefinger, the other three fingers fixed the dorsal skin individually and the little finger was used to fix the left hind leg of nude mice. A 3-mm incision was cut in the left groin and a 1-mm^3^ section of tumor tissue was inserted into the subcutaneous inguinal region to a depth of ∼5 mm. The inoculated mice were housed in a decontamination sterile barrier system at a constant temperature (25±2°C) and constant humidity (45–50%) and tumor formation and growth were recorded daily.

### Statistical analysis

Data are expressed as the mean ± SE. Statistics were determined using analysis of variance, the χ^2^ test or the Student’s t-test using SPSS 11.0 software (Windows). P<0.05 or P<0.01 were considered to indicate statistically significant differences.

## Results

### Percentage of CD133^+^ tumor cells for cell sorting

Flow cytometric analysis showed that the percentage of CD133^+^ glioblastoma cells in the total cell number was 2.31±0.57%, which complied with the immunomagnetic bead sorting system requirements for cell separation.

### Specific marker protein expression

**Figure 1:**
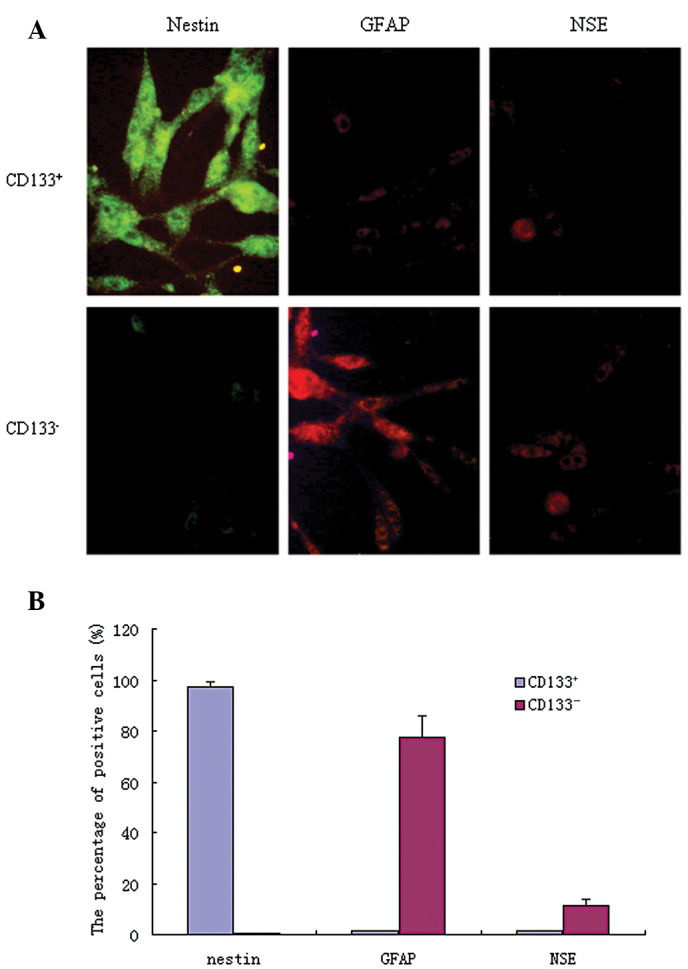
Specific marker protein expression of CD133^+/−^ glioblastoma cells. (A) Cells were stained with antibodies to nestin, GFAP or NSE by immunofluorescence assay; magnification, ×200. (B) Semi-quantitative bar graph of A. GFAP, glial fibrillary acidic protein; NSE, neuron-specific enolase.

As shown in Fig. 1, FITC-labeled antibodies appeared as green fluorescence, while TRITC-labeled antibodies appeared as red fluorescence. In CD133^+^ glioblastoma cells, the nestin-positive expression rate was 97.34±2.14%, GFAP was 1.44±0.27% and NSE was 1.35±0.24%. In CD133^−^ cells, the nestin-positive expression rate was 0.47±0.06%, GFAP was 77.41±8.49% and NSE was 11.38±2.21%. The expression levels of these molecules were significantly different between the CD133^+^ and CD133^−^ cells (P<0.05).

### Cell invasion assay

**Figure 2:**
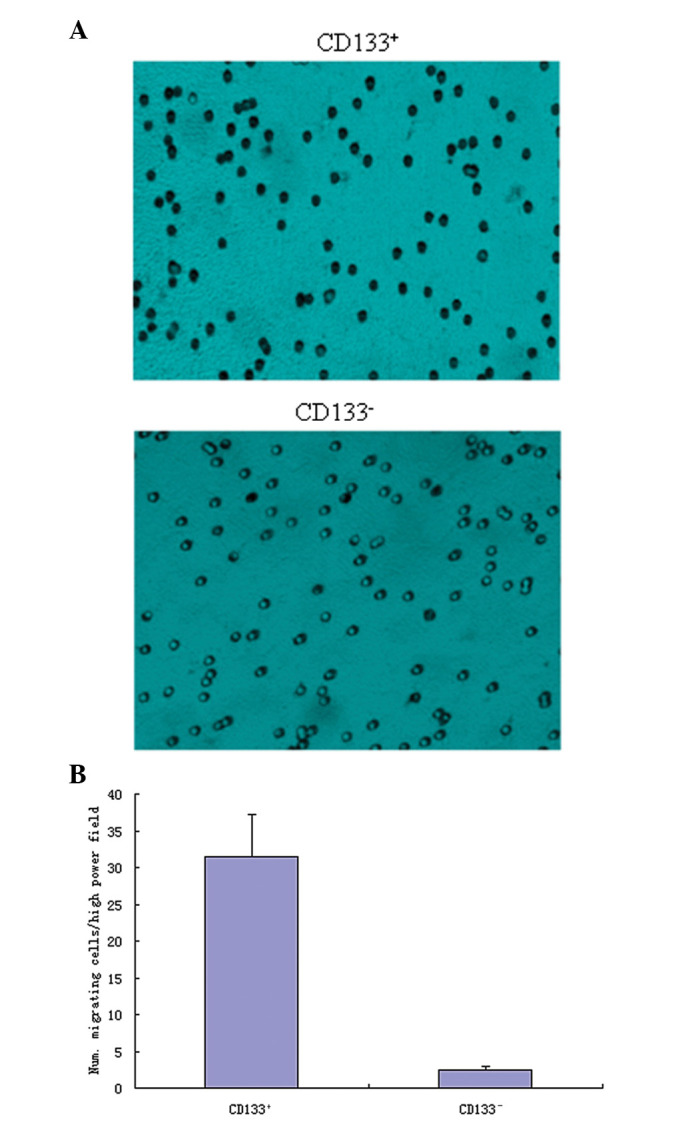
Comparison of CD133^+/−^ glioblastoma cell invasion ability. (A) In Transwell cell invasion experiments, invasive cells migrated to the lower chamber compared with non-migratory cells, which remained in the top chamber. Magnification, ×200. (B) Quantitative bar graph of A.

As shown in Fig. 2, the CD133^+^ glioblastoma cells showed a high invasive capacity, and had a significantly higher mean number of invasive cells in each high-power field (31.46±5.73), compared with the CD133^−^ cells (2.47±0.53; P<0.05).

### Serum dependence of tumor cells

**Figure 3:**
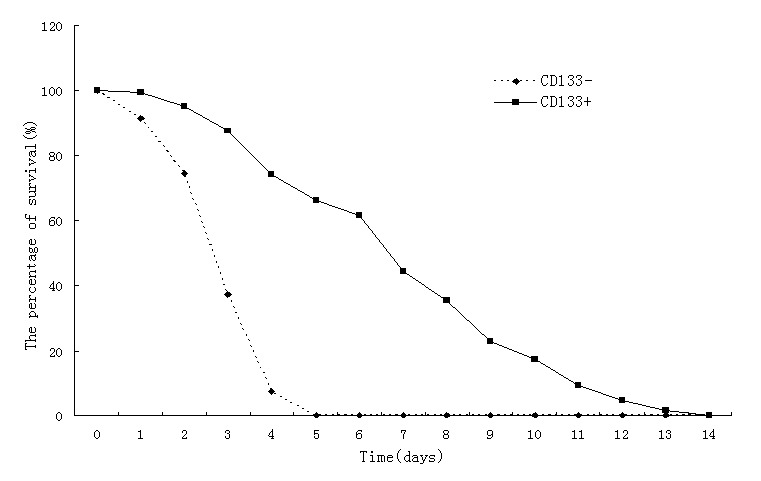
Comparison of the survival rate of CD133^+/−^ glioblastoma cells under low nutrient conditions. CD133^+^ glioblastoma cells have a greater tolerance to a low nutrient environment than CD133^−^ cells.

As shown in Fig. 3, the CD133^−^ glioblastoma cells did not survive beyond 5 days when grown in low serum conditions, while the CD133^+^ glioblastoma cells survived for up to 13 days under the same low serum culture conditions, which indicated a greater tolerance to a low nutrient environment.

### Soft agar colony formation experiment

**Figure 4:**
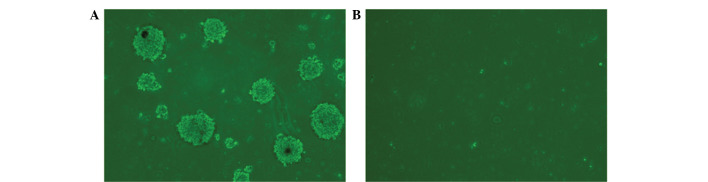
Comparison of clone formation capacity in CD133^+/−^ glioblastoma cells. CD133^+^ glioblastoma cells formed cell clones (A), while CD133^−^ glioblastoma cells did not (B). Magnification, ×200.

As shown in Fig. 4, 10 high-power fields of vision were randomly analyzed and the results revealed that the mean number of colonies forming >40 cells per high-power field in the CD133^+^ glioblastoma cell group was 7.18±1.17 compared with no colony formation in the CD133^−^ glioblastoma cell group.

### Comparison of the in vivo tumor formation rate

**Figure 5:**
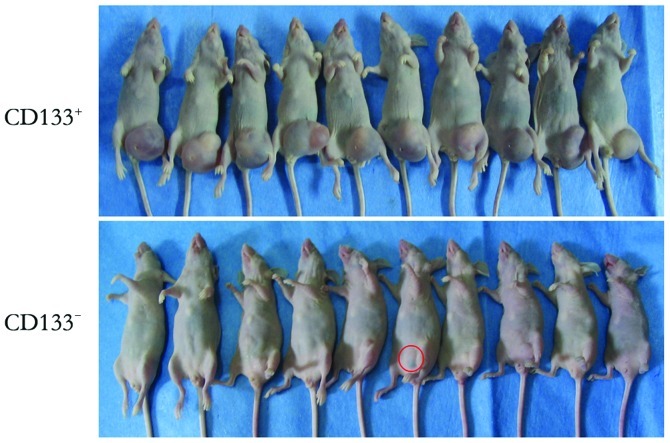
Subcutaneous tumor incidence of CD133^+/−^ glioblastoma cells transferred to thymectomized mice. The upper panel of mice indicates the tumor incidence of CD133^+^ glioblastoma cells *in vivo* (n=10/10). The lower line of mice indicates the CD133^−^ glioblastoma cell tumor incidence (n=1/10). The tumor formation is indicated by a red circle.

As shown in Fig. 5, the incidence of subcutaneous tumor formation in the thymectomized mice receiving CD133^+^ glioblastoma cells was 26/30 at 28 days post-transfer, compared with 2/30 in the CD133^−^ glioblastoma cell group. The CD133^−^ induced tumor volumes were also smaller.

### In situ apoptosis

**Figure 6:**
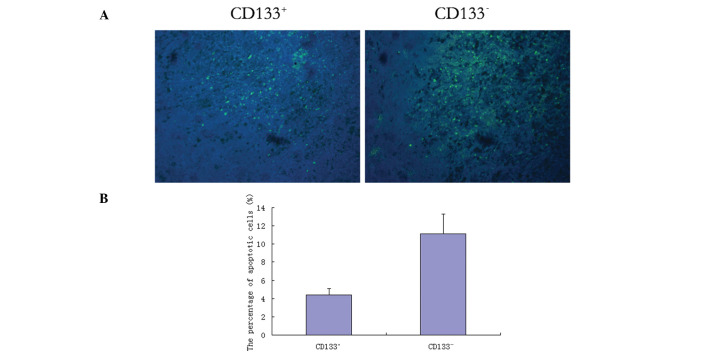
*In situ* apoptosis of CD133^+^ and CD133^−^ glioblastoma cell-induced tumor tissue. (A) Blue signal indicates fluorescence emitted from Hoechst 33258-labeled nuclei and the green signal indicates apoptotic cells. Magnification, ×100. (B) Semi-quantitative bar graph of A.

As shown in Fig. 6, the CD133^+^ glioblastoma cell-induced tumor tissue demonstrated scattered apoptotic cells (green stain). In 10 randomly selected high-power fields of vision, the apoptosis rate of CD133^+^ cells was significantly lower (4.37±0.74%) compared with that of CD133^−^ cells (11.14±2.15%; P<0.05).

### Western blot assay

**Figure 7:**
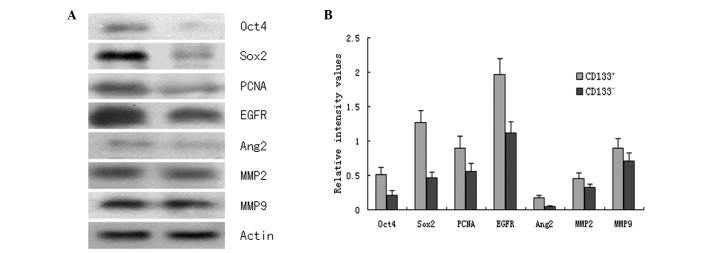
Comparison of protein expression levels in CD133^+/−^ glioblastoma cell-induced tumor tissues. (A) Western blot results; (B) semi-quantitative bar graph of A. There was a significant increase in protein expression in the CD133^+^ glioblastoma cell-induced tumor tissue compared with the CD133^−^ group (P<0.05).

As shown in Fig. 7, the CD133^+^ glioblastoma cell induced-tumor tissue expressed significantly higher levels of Oct4, Sox2, PCNA, EGFR, Ang2, MMP2 and MMP9 proteins compared with the CD133^−^ glioblastoma cell induced-tumor tissue (P<0.05).

### Flow cytometric detection

**Figure 8:**
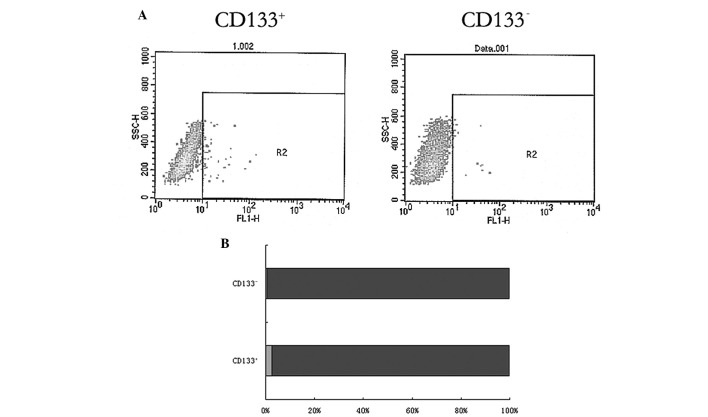
Percentage of CD133^+^ cells in the total number of tumor cells in CD133^+/−^ glioblastoma cell-induced recurrent tumors. (A) Flow cytometry assay results. (B) Quantitative bar graph of A.

As shown in Fig. 8, the R2 gate indicated the CD133^+^ glioblastoma cells. Flow cytometry detection showed that in the CD133^+^ glioblastoma cell-induced tumors, there was a significantly higher percentage of CD133^+^ cells (2.47±0.67% of the total cells) compared with the CD133^−^ glioblastoma cell-induced tumors (0.44±0.14% of total cells; P<0.05).

### In vivo transplantation tumor formation rate

The tumor formation rate was 10/10 in CD133^+^ glioblastoma cell-induced tumors following transplantation, but only 4/10 in CD133^−^ glioblastoma cell-induced tumor tissue. CD133^−^ tumor cell-induced recurrent tumor volumes were also smaller compared with the CD133^+^ cell-induced tumors.

## Discussion

In the present study, CD133^+/−^ tumor cells from the glioblastoma cells of 8 Han Chinese patients living in Northern China were obtained, and the biological characteristics of the cells were analyzed. Magnetic bead sorting is a commonly used technique for isolating tumor stem cells. Since CD133^+^ cells accounted for 0.3–2% of the total glioma cells (15–17), a low proportion does not favor immunomagnetic sorting. In this study, tumor specimens pathologically identified as type IV glioblastomas were used to increase the chance of a higher proportion of tumor cells for sorting. Flow cytometry analysis confirmed that CD133^+^ cells accounted for 2.31±0.57% of total cells in 8 cases of glioblastoma, which was consistent with the immunomagnetic bead sorting system requirements for cell separation. Not all tumor stem cells expressed CD133^+^, as the CD133^−^ cell population also contained some tumor stem cells. However, most CD133^+^ cells had high tumorigenicity and invasion characteristics (18) and therefore, CD133^+^ expressing glioblastoma cells were considered as the observation subject with the aim of further developing the scope and depth of understanding of glioma stem cell research.

Nestin is a type VI intermediate filament protein and its expression is restricted to central nervous system precursor cells, such as neural and glioma stem cells. Therefore, nestin may be used as a neural precursor cell marker (19). NSE occurs in mature neurons and may be used as a surface marker of mature neuronal cells (20). GFAP, a type III intermediate filament protein family member, is specifically expressed in astrocytes and thus is often considered to be an astroglial marker in neurobiological research (21). This study revealed that CD133^+^ glioblastoma cells expressed high levels of nestin but expressed low levels of GFAP and NSE. By contrast, CD133^−^ glioblastoma cells expressed low levels of nestin (0.47±0.06%), but had high expression levels of GFAP (77.41±8.49%) and NSE. This suggested that the majority of CD133^+^ glioblastoma cells are neural precursor cells, which rarely differentiate. Following 24 h of *in vitro* culture, CD133^−^ glioblastoma cells began to exhibit increases in cell body size, cytoplasmic light stain and a gradual extension of synapses. Immunofluorescence detected high levels of glial cells and neuron-specific marker protein expression which indicated differentiation to nerve cells.

A transwell dual-chamber culture system was used to detect the invasive capacity of the two types of tumor cells and revealed that CD133^+^ glioblastoma cells had higher cell invasion capacity than CD133^−^ cells. The serum dependence experiments compares the tolerance of less malignant or normal cell lines and highly malignant cells, such as tumor cell lines, to low nutrient conditions and the results are used as an indicator of survivability and to evaluate the degree of malignancy. The soft agar cell clone formation experiment was designed to mimic the semi-solid growth environment of the *in vivo* extracellular matrix and to determine *in vitro* cell colony growth potential. Tumor cells are capable of proliferating and show strong cloning ability while mature differentiated cells do not form colonies (22). The previous two experiments revealed that CD133^+^ glioblastoma cells had a greater tolerance to low nutrition and had a higher colony forming ability compared with CD133^−^ cells.

*In vivo* experiments demonstrated a significantly higher subcutaneous tumor incidence rate in the CD133^+^ group (26/30) compared with the CD133^−^ group (2/30) following inguinal subcutaneous inoculation of CD133^+^ or CD133^−^ glioblastoma cells into thymectomized mice. The *in vivo* experiments were repeated 3 times, but CD133^+^ cells did not induce tumors in all mice. Additionally, subcutaneous tumors formed in two mice inoculated with CD133^−^ cells. Therefore, the data suggest that CD133^+^ and CD133^−^ cells may be used as markers to distinguish tumor stem cells.

The present study also conducted histopathological analysis on 26 cases of CD133^+^ and 2 cases of CD133^−^ glioblastoma cell-induced subcutaneous tumors. Firstly, the tumor tissue was analyzed to detect *in situ* apoptosis which indicated that CD133^+^ cell-induced tumors had a low incidence of apoptosis compared with CD133^−^ glioblastoma cell-induced tumor cells. Secondly, quantitative expression of proteins in the two types of tumor tissues was determined. Oct4 and Sox2 are transcription factors expressed by stem cells and are involved in self-renewal (23,24). Oct4 belongs to the POU family, and maintains the undifferentiated state of embryonic stem cells and promotes their proliferation. Activation of Oct4 drives the reprogramming of somatic cells to pluripotent stem cells (25). Sox2 is a specific transcription factor expressed by embryonic stem cells and, similar to Oct4, is an essential gene for somatic cell reprogramming. Sox2 acts synergistically with Oct4 to regulate and maintain cellular pluripotent potential (26). In the present study, the expression of Oct4, Sox2 and PCNA in CD133^+^ glioblastoma cell-induced tumors was significantly higher than in CD133^−^ cell-induced tumors, indicating that CD133^+^ glioblastoma cell-induced tumors expressed higher levels of transcription factors to maintain pluripotent features and induce proliferation. EGFR drives the evolution of glioma malignancy through downstream signaling (27). Ang2 acts as an activator of multiple tumor migration factors and is important in glioma angiogenesis, matrix degradation and invasion (28). MMP2 and MMP9 protein expression levels are key indicators of glioma cell invasion (29,30). Notably, CD133^+^ glioblastoma cell-induced tumors expressed higher levels of EGFR, Ang2, MMP2 and MMP9 proteins compared with CD133^−^ cell-induced tumors, suggesting that CD133^+^ cell-induced tumors have a higher degree of malignancy. This evidence may explain the low recurrence of tumor formation following CD133^−^ cell inoculation into mice and the small tumor volume observed. However, the tumor recurrence rates following subcutaneous CD133^+^ and CD133^−^ cell transplantation were 10/10 and 4/10, respectively, which were higher than the tumor formation rates following the initial transfer of tumor cells (26/30 and 2/30 respectively), particularly for the CD133^−^-induced tumor cells. This suggests that recurrent tumors may have an increased degree of malignancy due to genetic change.

In the present study, flow cytometry was used to identify CD133 antigen-positive expression in tumor cells derived from two subtypes of cells, and demonstrated that in CD133^+^ cell-induced tumors, the percentage of CD133^+^ cells was 2.47±0.67%, similar to the CD133^+^ percentage obtained from human brain tumors during the initial surgery (2.31±0.57%). CD133^+^ tumor cells were also observed (0.44±0.14%) in the CD133^−^ cell-induced tumors. Therefore, CD133^−^ cells may differentiate into CD133^+^ cells and cells that do not express CD133 at the initial stages may express CD133 under certain environmental conditions. Thus, this study suggests that CD133 is not the only marker protein for the identification of glioma stem cells.

In summary, we successfully tested the biological characteristics of CD133^+^ and CD133^−^ glioblastoma cells of 8 Han Chinese individuals, verified the high tumorigenicity and invasiveness of CD133^+^ tumor cells and validated the limitations and deficiencies of the CD133 antigen as a marker to distinguish tumor stem cells. At present, research addressing human glioma stem cells is in the initial stages and consequently, it is vital to verify and further elucidate the methods of glioma stem cell isolation and identification.
